# Reduced graphene oxide enwrapped phosphors for long-term thermally stable phosphor converted white light emitting diodes

**DOI:** 10.1038/srep33993

**Published:** 2016-09-27

**Authors:** Gopinathan Anoop, Janardhanan R. Rani, Juhwan Lim, Myoung Soo Jang, Dong Wook Suh, Shinill Kang, Seong Chan Jun, Jae Soo Yoo

**Affiliations:** 1 School of Chemical Engineering and Materials Science, Chung-Ang University, Heukseok-Dong 221, Dongjak-gu, Seoul-06974, Republic of Korea; 2 School of Materials Science and Engineering, Gwangju Institute of Science and Technology, Gwangju-61005, Republic of Korea; 3 School of Mechanical Engineering, Yonsei University, 50 Yonsei-ro, Seodaemun-gu, Seoul-03722, Republic of Korea

## Abstract

The long-term instability of the presently available best commercial phosphor-converted light-emitting diodes (pcLEDs) is the most serious obstacle for the realization of low-cost and energy-saving lighting applications. Emission from pcLEDs starts to degrade after approximately 200 h of operation because of thermal degradation of the phosphors. We propose a new strategy to overcome this thermal degradation problem of phosphors by wrapping the phosphor particles with reduced graphene oxide (rGO). Through the rGO wrapping, we have succeeded in controlling the thermal degradation of phosphors and improving the stability of fabricated pcLEDs. We have fabricated pcLEDs with long-term stability that maintain nearly 98% of their initial luminescence emission intensity even after 800 h of continuous operation at 85 °C and 85% relative humidity. The pcLEDs fabricated using SrBaSi_2_O_2_N_2_:Eu^2+^ phosphor particles wrapped with reduced graphene oxide are thermally stable because of enhanced heat dissipation that prevents the ionization of Eu^2+^ to Eu^3+^. We believe that this technique can be applied to other rare-earth doped phosphors for the realization of highly efficient and stable white LEDs.

In solid state lighting technology, the most widely accepted method for white light generation is to use a blue GaN-based or InGaN-based light-emitting diode (LED) with a conversion phosphor (embedded in epoxy resin/binder) that partially converts blue light into yellow light. The resulting combination of yellow light with the residual blue light produces white light as perceived by the human eye^1^,^2^,^3^,^4^,^5^. These phosphors should efficiently down-convert the blue photon and should maintain good optical performance even at an elevated ambient temperature. Even though GaN/InGaN-based LEDs have a very long lifetime (~50,000–100,000 h), the light emission from phosphor-converted LEDs (pcLEDs) degrades over time with an average lifetime of only about 7000–9000 h. The major reasons for the degradation of pcLEDs are emission intensity reduction from phosphors, which is predominantly due to thermal quenching, and thermal degradation^6^. Thermal quenching of luminescence is the result of excess heat at the p-n junction, which results in an increase in the probability of non-radiative transitions and consequently degrades the efficiency of the phosphor^2^,^4^. Thermal degradation results from the thermal/photo oxidation of activator ions in the phosphor^2^,^4^. Usually, the excess heat that is generated at the p-n junction of a blue LED does not dissipate through the phosphor layer, as these inorganic phosphors have very low thermal conductivity. Most commercial LED phosphors that have high conversion efficacy are susceptible to either thermal quenching or thermal degradation, which are serious issues that limit the lifetime of pcLEDs^1^,^7^,^8^. Because of this, only a limited number of phosphors–those that are most resistant to thermal quenching or thermal degradation–find application in commercial pcLEDs, even though a large number of phosphors with high conversion efficacy are available.

One of the strategies to extend the lifetime of pcLEDs is to reduce thermal degradation of phosphors through dissipating the excess heat generated at the junction of blue LED. This would offer a wider choice among the available phosphors that show excellent conversion efficacy and would extend the lifetime of pcLEDs. One possible way of thermal management in pcLEDs is wrapping the conversion phosphors using materials having high thermal conductivity and high optical transparency. However, it is challenging to design suitable materials that are thermally conducting and optically transparent to visible light.

Owing to its high thermal conductivity, optical transparency, and chemical inertness, graphene and reduced graphene oxide (rGO) are possible candidates for thermal management applications^9^,^10^,^11^. An unusually high thermal conductivity (~5300 W/mK) at room temperature has been obtained for single layer graphene^12^. While the thermal conductivity of rGO varies with the percentage of oxygen functional groups attached on to the hexagonal carbon matrix, through control of the oxygen functional groups, it can be made to exhibit a thermal conductivity of up to 53% of that of the pristine graphene^13^. Owing to the ease of solution processability, rGO is a good thermal management material in electronic and optoelectronic devices, especially, where the temperature plays an important role in the degradation or poor performance of the devices^14^. Improved heat dissipation has been achieved for GaN LEDs with graphene^15^, graphene-oxide (GO)^14^ and carbon nanotubes (CNT)-graphene hybrid embedded in sapphire substrate^16^. However, such techniques not only require complex experimental procedures but also alter the single crystalline nature of the sapphire substrate.

Here, we introduce a simple technique, using conventional solid-state synthesis, for thermal management of pcLEDs fabricated with the phosphor (SrBa)Si_2_O_2_N_2_:Eu^2+^. The phosphor particles were wrapped with GO by mixing with an appropriate amount of GO, followed by annealing at various temperatures. Our study shows that annealing the GO-phosphor composites at high temperature (≥1200 °C) results in the reduction of GO to rGO followed by a wrapping of rGO around the phosphor particles. We find that rGO wrapping serves as an excellent thermal management material, and pcLEDs fabricated in this manner exhibit long-term reliability, unlike pcLEDs fabricated with unwrapped phosphor.

## Results and Discussion

**Figure 1:**
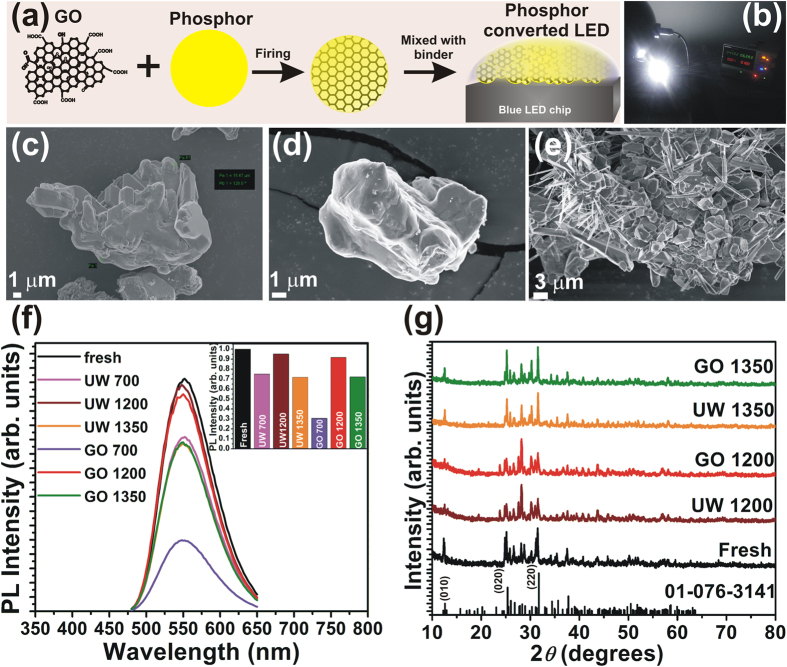
(**a**) Schematic illustration showing experimental method of GO wrapping over phosphor. (**b**) Digital image of the fabricated pcLED using GO 1200 phosphor operated at 100 mA. (**c–e**) FESEM images of fresh phosphor and phosphor samples GO 1200, and GO 1350. (**f**) PL of the fresh, annealed, and GO-wrapped phosphors. Inset shows the variation in PL intensity with annealing and GO wrapping. (**g**) XRD patterns of fresh, annealed, and GO-wrapped phosphors.

The rGO wrapping was achieved through mixing appropriate amount of GO and phosphor followed by a calcination at temperatures of 700 °C, 1200 °C, and 1350 °C for 6 h in a reducing (N_2_/H_2_) atmosphere. The annealing of GO-phosphor composite results in a wrapping of rGO around the phosphor particles. Figure 1(a) shows a schematic illustration of the synthesis of rGO wrapped phosphor. The samples of unwrapped phosphors annealed at 700, 1200 and 1350 °C are labelled as UW 700, UW 1200 and UW 1350 while those annealed with GO are labeled as GO 700, GO 1200 and GO 1350 respectively. Figure 1(b) shows digital image of the pcLED operated at 100 mA current.

In order to confirm that the GO was wrapped around the phosphor particles, field emission scanning electron microscope (FESEM) images were analyzed. Micrographs of the phosphors annealed with and without GO are shown in Fig. 1(c–e). Figure 1(c) is an image of a fresh phosphor particle. An FESEM image of a GO 1200 phosphor particle is shown in Fig. 1(d), and it is apparent that there is a change in contrast of the particle surface, apparently due to the rGO wrapping. Interestingly, for the GO 1350 phosphor sample (Fig. 1(e)), a number of rod-like structures are observed. These rod structures are nothing but rGO sheets that are transformed in to nanoscrolls. This scrolling is a result of the interaction of heavy phosphor particles with rGO sheet during annealing which results in an extremely unfavorable condition for rGO to maintain its planar structure^17^,^18^. During the annealing of the GO-phosphor composite at high temperatures, along with the removal of functional groups, the phosphor particles are also attached to the rGO sheets. When the thermal energy is high enough, the interaction of phosphor particles with rGO sheets leads to bending of the rGO sheets, in order to reduce the surface energy of the rGO-phosphor system. This is because, the interaction between phosphor particles and rGO renders the planar structure of the rGO sheets extremely unfavorable^19^. The bending of the rGO sheets is followed by rolling to form scrolls because of Van der Waals attraction and the π-π stacking effect between rGO layers^17^,^18^. The formation of the nanoscrolls occurs in two steps. First, the rGO sheets attach to the phosphor particles. When the temperature increases, the sheets bend around the phosphor particles in order to reduce the surface energy of the system. This bending results in wrapping of rGO sheets around the phosphor particles as observed in GO 1200. Second, when the temperature is further increased after the phosphor is enwrapped by a few layers of rGO, it becomes difficult for the sheets to maintain their flat 2D structure because of the additional thermal energy. In order to reduce the total surface energy of the rGO-phosphor system, rolling is triggered, resulting in the formation of scrolls.

Additional SEM images of unwrapped phosphor particles as well as GO 1200 and GO 1350 phosphor particles are shown in the Supplementary Information in Figs S1 and S2 respectively. From the SEM images, we conclude that in sample GO 1200 most of the phosphor particles are wrapped by rGO whereas in GO 1350 because of scroll formation, a complete wrapping of phosphor particles does not occur.

Figure 1(f) shows photoluminescent (PL) emission from fresh, unwrapped, and rGO-wrapped phosphors. Compared to fresh phosphor, the PL emission intensity decreases substantially for the UW 700, UW 1350, GO 700, and GO 1350 samples. However, the PL emission intensity for UW 1200 and GO 1200 phosphors is nearly 95% of that of fresh phosphor. The PL intensity for the GO 700 samples decreased to 30% of that of fresh phosphor. This is presumably because of the wrapping of unreduced or poorly reduced GO that possesses a relatively high amount of oxygen functional groups that limit light emission. The presence of these oxygen functional groups results in some absorption of both incident and emitted light. This considerably reduces the overall luminescent emission intensity from the phosphor. In GO 1200 and GO 1350, there are fewer oxygen functional groups compared to GO 700 and PL emission intensity from the GO 1200 phosphor samples remains nearly as high as that of fresh samples.

In order to confirm the reduction of oxygen functional groups, C *1s* x-ray photoelectron spectroscopy (XPS) data from pure GO, GO 1200, and GO 1350 were recorded and analyzed. The spectra are shown in Supplementary Fig. S3. In order to understand the nature of bonding between phosphor particles and rGO, the Sr *3d*, Si *2p*, O *1s*, and N *1s* high resolution XPS spectra (Supplementary Fig. S4) of unwrapped and wrapped phosphors were also analyzed. It is apparent from the spectra that the binding energy peaks related to oxygen functional groups reduce significantly for GO 1200 and GO 1350 compared to pure GO. At 1200 °C, reduction of GO occurs making the wrapping more transparent to visible light and allowing better transmission of the light incident on and emitted from the phosphor. The removal of oxygen functional groups was also analyzed using Fourier transform infrared (FTIR) spectroscopy. The FTIR spectra of the GO, fresh phosphor, GO 1200, and GO 1350 are shown in Supplementary Fig. S5. The FTIR data also confirm the reduction of GO through annealing. The stability of GO at higher temperatures was also analyzed using thermogravimetric analysis (TGA) in N_2_ atmosphere. Figure S6 shows TG thermograms for GO, GO-phosphor, and fresh phosphor. GO exhibits around 68% weight loss at 1350 °C whereas no significant weight loss is observed in the thermogram of fresh phosphor. The x-ray diffraction (XRD) patterns of the fresh phosphor and the phosphor annealed with and without GO are shown in Fig. 1(g). The patterns match well with the standard PDF 01-076-3141, and no additional peaks related to the constituent reactants were observed. It should be noted that the GO wrapping does not cause any significant variation in the crystal structure of the phosphor.

**Figure 2:**
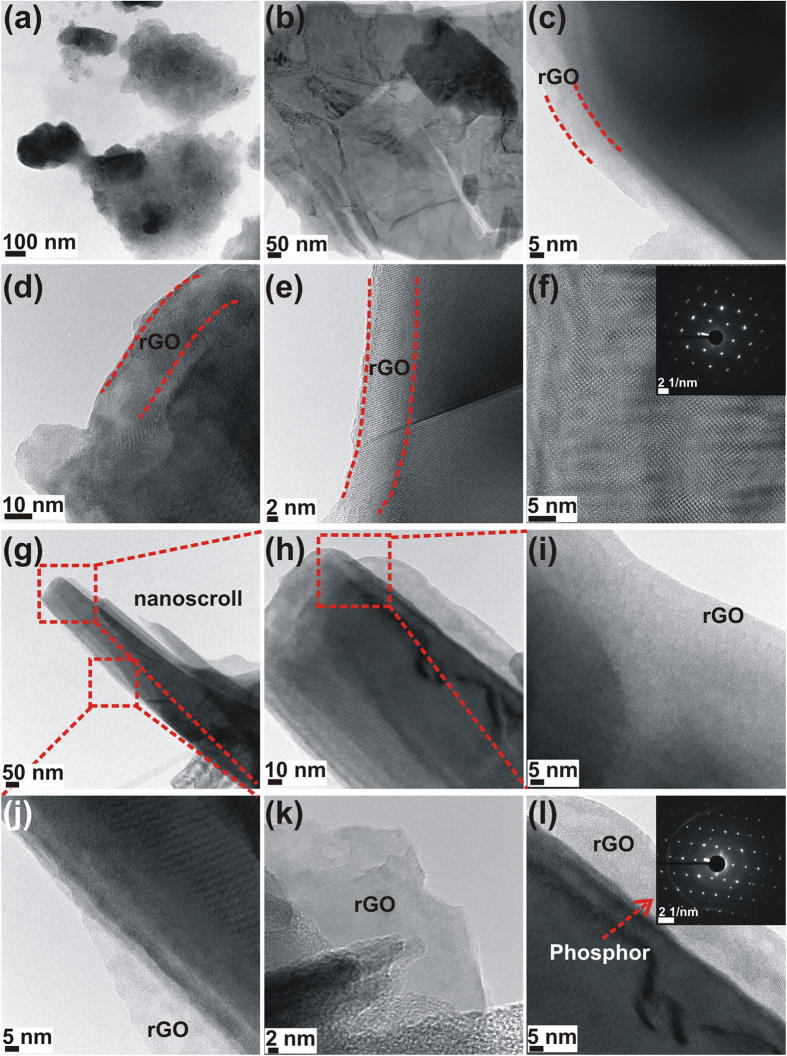
(**a–f**) TEM and HRTEM images of GO 1200, (**g–l**) TEM and HRTEM images of GO 1350. The wrapping of rGO over GO 1200 phopshors is clearly visibile, while in GO 1350, the self-wrapping of rGO sheets results in the formation of nanoscrolls.

For samples GO 1200 and GO 1350, we investigated the crystalline nature of the rGO and further characterized its wrapping of the phosphor particles using transmission electron microscopy (TEM) and high-resolution transmission electron microscopy (HRTEM). Figure 2(a–f) are HRTEM images from sample GO 1200 and Fig. 2(g–l) are images from sample GO 1350. Figure 2(a,b) are images of phosphor particles that are wrapped in rGO. Figure 2(c–e) confirm that the phosphor particles are wrapped with several layers of rGO sheets. Figure 2(f) is an HRTEM image of rGO sheets, which exhibits lattice fringes indicative of the honeycomb lattice. The selected area electron diffraction (SAED) pattern from this section is shown in the inset of Fig. 2(f), and it confirms the hexagonal symmetry and highly ordered nature of the rGO. Only six spots are observed in the inner hexagon of the SAED pattern, which can be indexed as a single crystal of AB Bernal stacked graphite^20^. The *d* value calculated from the spacing of HRTEM images is 0.37 nm, which is slightly high compared to the standard value of 0.34 nm for (0002) plane spacing. This is presumably because of the presence of a small percentage of oxygen functional groups.

Some double or split spots are observed in the SAED pattern; these may arise because of back-folding of edges, overlapping domains, or intrinsic rotational stacking faults^20^. For sample GO 1350, the TEM image in Fig. 2(g) and HRTEM images in Fig. 2(h–l) clearly confirm the formation of nanoscrolls. The SAED shown in the inset of Fig. 2(l) corresponds to the diffraction pattern from the phosphor.

**Figure 3:**
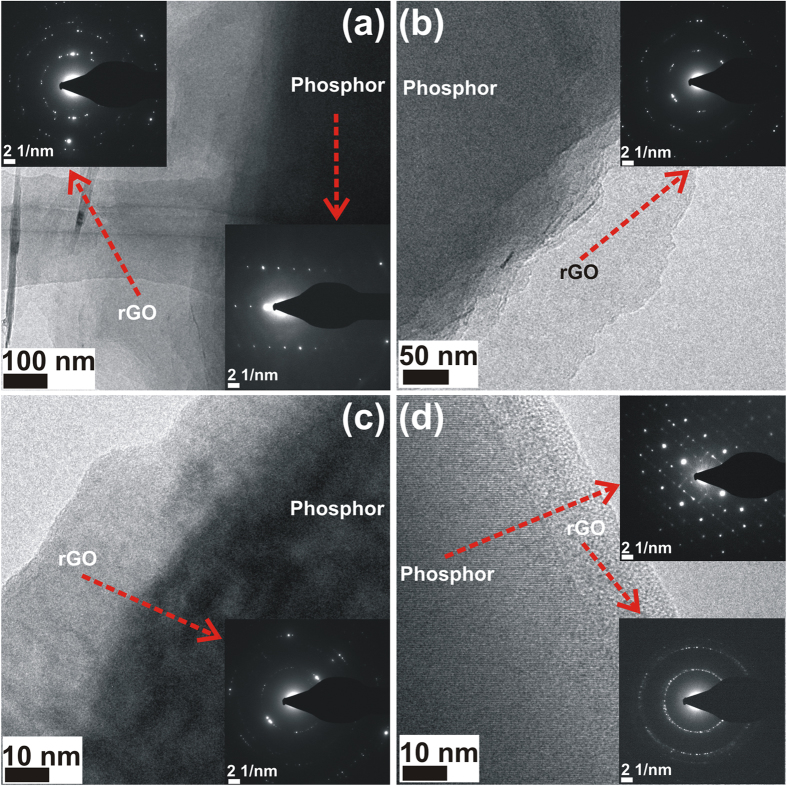
TEM and HRTEM images of GO 1350 from various regions. The corresponding SAED patterns are also shown in the inset.

Additional TEM/HRTEM images of GO 1350 phosphor samples are depicted in Fig. 3(a–d). In all the images, a contrast variation is observed at the edges of the images which are apparently rGO nanoscrolls wrapped over the phosphor particles. In order to confirm this, the diffraction patterns are recorded from the edges and the inner regions of the rGO wrapped phosphor particle. The SAED patterns are shown in the inset of Fig. 3(a–d) and corresponding regions from which the patterns are recorded, is also marked as red arrows. A clear difference in the diffraction patterns is observed for those recorded from the rGO (edges) as well as from the phosphor regions (inner region) of the rGO wrapped phosphor particles. The SAED pattern recorded from the inner regions correspond to the phosphor^21^ while the SAED pattern recorded from edges correspond to rGO^22^,^23^,^24^.

**Figure 4:**
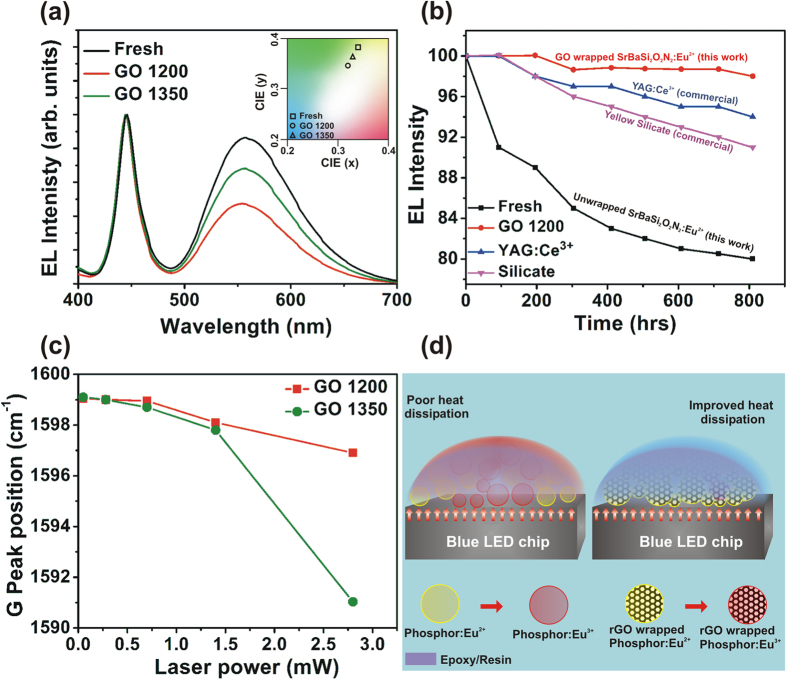
(**a**) EL spectra of the pcLEDs fabricated using fresh, GO 1200 and GO 1350 phosphors. The inset shows the corresponding CIE coordinates. (**b**) Variation of EL intensities with time of pcLEDs fabricated using fresh and GO 1200 phosphors as well as commercial YAG:Ce^3+^ and silicate phosphor and operated at 85 °C and 85% rel. humidity. The long term reliability test under stress conditions shows superior thermal stability of rGO wrapped phosphors compared to fresh and commercial phosphors. (**c**) The Raman G peak shift as a function of the incident laser power. (**d**) Schematic illustration showing the heat dissipation mechanism in pcLEDs fabricated using unwrapped and rGO-wrapped phosphor. Because of the high thermal conductivity of rGO, thermal oxidation of Eu^2+^ activators to Eu^3+^ is considerably reduced, which gives rise to long term reliability of pcLEDs fabricated using rGO-wrapped phosphors.

Since the GO 700 phosphor exhibits very low PL emission intensity, only fresh phosphor, GO 1200, and GO 1350 phosphors were used for fabricating pcLEDs. The electroluminescent (EL) spectra from the pcLEDs fabricated from fresh phosphor and from phosphors GO 1200 and GO 1350 are shown in Fig. 4(a). The corresponding commission internationale d’Eclaraige (CIE) coordinates are also shown in the inset of Fig. 4(a). A minor shift in the coordinates is observed, presumably due to the rGO wrapping. If there is a large shift in the CIE coordinates, the quality of the emitted white light from the pcLEDs will be poor^2^. It is worth mentioning that in our rGO wrapped phosphors, shift in the CIE coordinates is trivial and therefore rGO wrapping does not affect the quality of emitted light. The relative EL intensity from the phosphor (yellow region of the spectrum) decreases for the LEDs fabricated using GO 1200 and GO 1350 phosphors presumably because of the rGO coating on the phosphor particles. The optical parameters of the fabricated LEDs are summarized in Table 1. The luminescence efficacy of the device fabricated using fresh phosphor was 117 lm/W while that of the device fabricated using GO 1200 was 98 lm/W. The efficiency drop corresponds to the reduced emission intensity because of absorption of emitted light in the rGO wrapping. However, the color rendering index (CRI) values are relatively high for GO 1200 and GO 1350. Among all the fabricated pcLEDs, the one made with GO 1200 phosphor exhibited the highest CRI Ra of 70 (Table 1).

The reliability of the phosphor was tested by operating the LEDs continuously in a stress chamber of 85 °C and 85% relative humidity. Figure 4(b) is a plot of the EL intensity as a function of operating time for pcLEDs fabricated with GO 1200 and with unwrapped phosphor. In addition, data from the pcLEDs fabricated using two commercially available phosphors are shown. The reliability test plot of the pcLEDs fabricated using all the phosphors are shown in Supplementary Fig. S7. The pcLEDs fabricated using GO 1200 phosphor show enhanced stability when compared to other samples, which is because of the enhanced heat dissipation arising from a complete wrapping of phosphor particles by rGO. For the pcLEDs fabricated with unwrapped phosphor, the reduction of EL intensity over time is caused by thermal oxidation of Eu^2+^ ions to Eu^3+^ in the host lattice. This significantly reduces the EL emission intensity, and, after 800 h of continuous operation at 85 °C and 85% relative humidity, the emission drops to 80% of the initial value. This oxidation/auto ionization is considerably reduced through rGO wrapping, as the heat generated at the p-n junction in the LEDs is effectively conducted away by rGO sheets which are wrapped around the phosphor particles. Several issues lead to the degradation of the white light emission from the pcLEDs including thermal quenching of the phosphor, thermal degradation, thermal/photo oxidation, and moisture-induced oxidation^2^,^6^,^25^. It should be noted that each phosphor behaves differently under these stress conditions depending on the crystal structure, phosphor particle size, and most importantly its thermal and humidity resistance.

In the present study, since the pcLEDs are operated continuously for 800 h, heat generated at the junction of the blue LED will raise the temperature above the ambient 85 °C, thereby accelerating the thermal degradation. The excess heat is transferred to the phosphor via conduction, but, because of the low thermal conductivity of the phosphor layer, the heat is not efficiently conducted away from the device. This excess heating results in thermal oxidation of the host phosphor (SrBaSi_2_O_2_N_2_) as well as the Eu^2+^ activator. The extent of oxidation depends on the stability of the crystal structure of the phosphor. If the phosphor particles have cracks or the crystal structure of the phosphor consists of several voids, then the oxidant gases can penetrate easily and oxidize the phosphor. In some phosphors, such penetration results in the formation of a second phase that considerably degrades the luminescent emission^6^. From our study, it is apparent that the commercial phosphors are much more resistant to oxidation compared to our unwrapped SrBaSi_2_O_2_N_2_:Eu^2+^ phosphor. On the other hand, rGO-wrapped SrBaSi_2_O_2_N_2_:Eu^2+^ phosphor exhibit better oxidation resistance due to the conduction of excess heat through rGO layers. This is why the luminescence emission from the fresh phosphor decreases very rapidly over time while the rGO-wrapped phosphor exhibits a stable emission. Apart from the excess heat, moisture also plays a significant role in thermal degradation. The presence of moisture along with the excess heat accelerates oxidation. The presence of moisture also plays a role in oxidizing the rGO, which then reduces thermal conductivity. Moreover, the oxidation results in the formation of oxygen related functional groups that lead to increased absorption of emitted light by π electrons in rGO and, consequently, reduced luminescent emission. This could be one of the reasons for the degradation of emission intensity after 200 h of continuous operation of rGO wrapped LEDs. More studies are underway to understand the exact reasons for the degradation of our pcLEDs after 200 h of operation.

In order to confirm the heat dissipation through rGO wrapping, we performed Raman spectroscopy at various laser powers and analyzed the shift of G peak at various laser powers. The experimental details of the measurement can be found in a previous report^26^. Raman spectrometry acts as a thermometer measuring the local temperature rise in graphene in response to the Raman laser heating. Graphene has a distinctive signature in its Raman spectrum with a clear temperature-dependent G peak. It is known that the position of the G peak exhibits strong temperature dependence; the peak redshifts with an increase in temperature^12^,^26^,^27^,^28^,^29^. In the present study, GO 1200 and GO 1350 samples were heated using the focused Raman laser beam at different powers. If there is an increase in the temperature of the samples through a focused laser excitation, it will lead to a red shift of the G peak. This is because of bond softening; a rise in local temperature causes thermal expansion or anharmonic coupling of phonon modes, which is caused by elongation of the C-C bond^12^. Figure 4(c) shows the shift of G peak as a function of the incident laser power in GO 1200 and GO 1350 phosphors. From the figure, it is clear that the red shift of the G peak is negligible for GO 1200 at lower laser powers and a minor shift is observed at a higher power. This demonstrates better heat transfer/conduction from the sample; heating by the focused laser is well dissipated from the sample. It is apparent from the plot that, for a higher laser power, the shift of the Raman G peak is much smaller in GO 1200 than GO 1350. This clearly suggests that a much better heat dissipation from GO 1200 results in a lower temperature rise than in GO 1350. A better heat conduction results in subduing the thermal degradation of phosphors leading to the longevity of the fabricated pcLEDs. As observed in FESEM and TEM/HRTEM images, the self-wrapping of rGO sheets leading to nanoscroll formation reduces the heat dissipation in the phosphor, which results in a poor thermal management in pcLEDs fabricated with the GO 1350 phosphor. This explains the long-term reliability of GO 1200 compared to GO 1350 (Fig. S7). In order to confirm the heat dissipation, the Raman spectra of GO 1200 and GO 1350 phosphors were also recorded at different temperatures. The Raman spectra of GO 1200 and GO 1350 at room temperature, 50, 85, and 120 °C are shown in Supplementary Fig. S8. A larger shift of the G peak position in GO 1350 confirms poor heat dissipation in GO 1350 phosphors. In conclusion, the excellent stability of graphene-coated phosphor particles is because of better heat dissipation achieved through complete rGO wrapping around the phosphor particles in GO 1200 sample.

Thus, Raman spectra confirm that in our pcLEDs with GO-wrapped phosphor particles, the heat generated at the junction is dissipated through rGO because of the higher thermal conductivity of rGO. In the pcLEDs with unwrapped phosphor particles, the low thermal conductivity of the phosphor suppresses heat transport resulting in the auto ionization of Eu^2+^ to Eu^3+^. A schematic illustration explaining the heat dissipation in fabricated LEDs with and without GO wrapping of phosphors is shown in Fig. 4(d). Compared to the GO 1200 sample, GO 1350 shows a reduction in EL intensity and poor long-term stability. As discussed earlier, in GO 1350 the GO sheets form nanoscrolls hindering complete wrapping of the phosphor particles. This reduces the thermal conduction resulting in poor reliability compared to that of GO 1200.

## Conclusions

In summary, the thermal degradation in pcLEDs has been effectively controlled through wrapping of rGO around phosphor particles using a simple solid-state reaction. There exists an appropriate annealing temperature to effectively achieve this wrapping. At higher temperatures, the phosphor particles interact with the rGO causing the rGO sheets to form nanoscrolls. In our experiment, we used a SrBaSi_2_O_2_N_2_:Eu^2+^ phosphor and found that 1200 °C was an appropriate temperature for complete wrapping of phosphor particles with rGO. We operated the pcLEDs fabricated using rGO wrapped phosphors continuously for 800 h at 85 °C and 85% relative humidity, and they maintain emission intensity at 98% of the initial value. The pcLEDs fabricated using unwrapped phosphors showed significant thermal degradation, and their emission intensity dropped to 80% of the initial value. Because of improved heat dissipation through the wrapping of rGO sheets around the phosphor particles, the oxidation of Eu^2+^ ions to Eu^3+^ is prevented, leading to better stability in rGO-wrapped phosphor-based pcLEDs. It is expected that our rGO wrapping technique can be extended to fabricate pcLEDs using other LED phosphors with high efficiency where the thermal-management-related instability is a major hurdle in realizing pcLEDs with long operation stability.

## Materials and Methods

The phosphors were synthesized using a high temperature solid-state reaction of the constituent components: SrCO_3_, BaCO_3_ (Kojundo 99.999%), Si_3_N_4_ (Ube, 99.99%), and Eu_2_O_3_ (Alfa Aesar, 99.99%). The powders were mixed stoichiometrically, ball milled in an acetone medium, and calcined at 1350 °C for 6 h in a N_2_/H_2_ (95:5) atmosphere to get Sr_1−x_Ba_x_ Si_2_O_2_N_2_:Eu (x = 0.40). Graphite oxide, synthesized by a modified Hummers method, was exfoliated to give a brown dispersion of graphene oxide (GO) under ultrasonication. Dispersions of GO with concentrations ranging from 0.5 mg/ml to 1 mg/ml were prepared. The phosphor was mixed with GO solution and was stirred continuously for 3 h using a magnetic stirrer. Samples of mixed GO-phosphor composite and of fresh phosphor (not mixed with GO) were annealed at temperatures of 700 °C, 1200 °C, and 1350 °C for 6 h in a reducing atmosphere (N_2_/H_2_). The crystal structure of the phosphors was analyzed using powder X-ray diffraction with Cu K_α_ radiation (Bruker, D8 Advance) and high-resolution transmission electron microscopy (HRTEM) (JEOL JEM 2100F). The thermogravimetric analysis (TGA) was carried out using SDT Q600 (TA Instruments, USA). FTIR measurements were carried out using Fourier transform infrared Nicolet 6700 Spectrometer (Thermoscientific, USA) with KBr as reference. The room temperature photoluminescent emission (PL) and excitation (PLE) spectra were recorded using a computer-controlled photomultiplier tube (PMT) and a xenon lamp (PSI, Korea). X-ray photoelectron spectroscopy (XPS) spectra were recorded using a ULVAC PHI 5000 Versaprobe with an Al anode. Phosphor-converted white LEDs were fabricated using yellow Sr_0.53_Ba_0.4_Si_2_O_2_N_2_:Eu_0.07_ phosphor and blue LED chips. The phosphors were mixed with silicone binder in appropriate weight ratios and finally deposited over blue InGaN chips using a conventional phosphor dispensing method. In the present work, phosphor-silicone binder mixture was dispensed over a double-cup 6030 InGaN blue LED. The electroluminescent (EL) characteristics were measured using a computer-controlled CCD detector and an integrating sphere (PSI, Korea) that was pre-calibrated using a standard white light source. The structural variations of the rGO wrapped phosphors were analysed using a Raman spectroscopy (Labram-HR, Horiba Jobin Yvon). The Raman spectra were also recorded at various laser powers as well as heating the phosphor samples at 50, 85, and 120 °C.

## Additional Information

**How to cite this article**: Anoop, G. *et al*. Reduced graphene oxide enwrapped phosphors for long-term thermally stable phosphor converted white light emitting diodes. *Sci. Rep.* **6**, 33993; doi: 10.1038/srep33993 (2016).

## Supplementary Material

Supplementary Information

## Figures and Tables

**Table 1:** Optical properties of fabricated white LEDs using unwrapped and wrapped phosphors.

Phosphor	CIE x	CIE y	CCT$ (K)	Efficacy (lm/W)	CRI# Ra
Fresh	0.3404	0.3779	5263	117	59.5
UW 1200	0.3201	0.3372	6077	108	63.4
UW 1350	0.3302	0.3546	5594	94	65.6
GO 1200	0.3186	0.3472	6109	98	70
GO 1350	0.3246	0.3543	5827	89	62
Silicate (commercial)	0.3143	0.3375	6370	165	59.1

^$^CCT-coordinated color temperature. CCT values provide a general indication of the apparent “warmth” or “coolness” of the light emitted by the source.

^#^CRI-color rendering index.
