# Supplementary material for: Reduced graphene oxide enwrapped phosphors for long-term thermally stable phosphor converted white light emitting diodes
Source: Sci Rep. 2016 Sep 27;6:33993. doi: 10.1038/srep33993 (PMC5037423; doi:10.1038/srep33993)
Supplement: Supplementary Information [file srep33993-s1.pdf]

Supplementary Information

Reduced graphene oxide enwrapped phosphors for long-term thermally stable phosphor converted white light emitting diodes

Gopinathan Anoop[a,c], Janardhanan R. Rani[b], Juhwan Lim[b], Myoung Soo Jang[a], Dong Wook Suh[a], Shinill Kang[b], Seong Chan Jun[b], and Jae Soo Yoo[a*]*

[a]School of Chemical Engineering and Materials Science, Chung Ang University, Heukseok-Dong 221, Dongjak-gu, Seoul-06974, Republic of Korea.

[b]School of Mechanical Engineering, Yonsei University, 50 Yonsei-ro, Seodaemun-gu, Seoul-03722, Republic of Korea.

[c]School of Materials Science and Engineering, Gwangju Institute of Science and Technology, Gwangju-61005, Republic of Korea.

***jsyoo@cau.ac.kr; scj@yonsei.ac.kr**

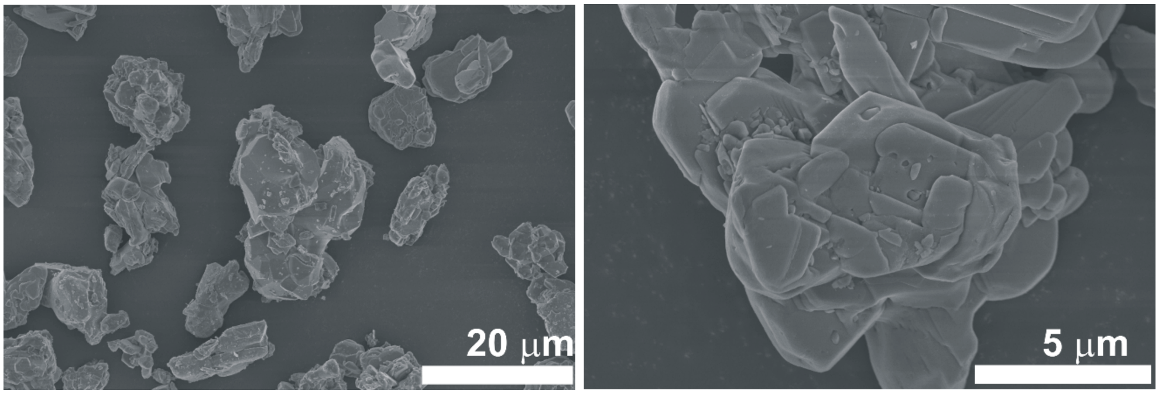

Figure S1. SEM images of Fresh phosphor

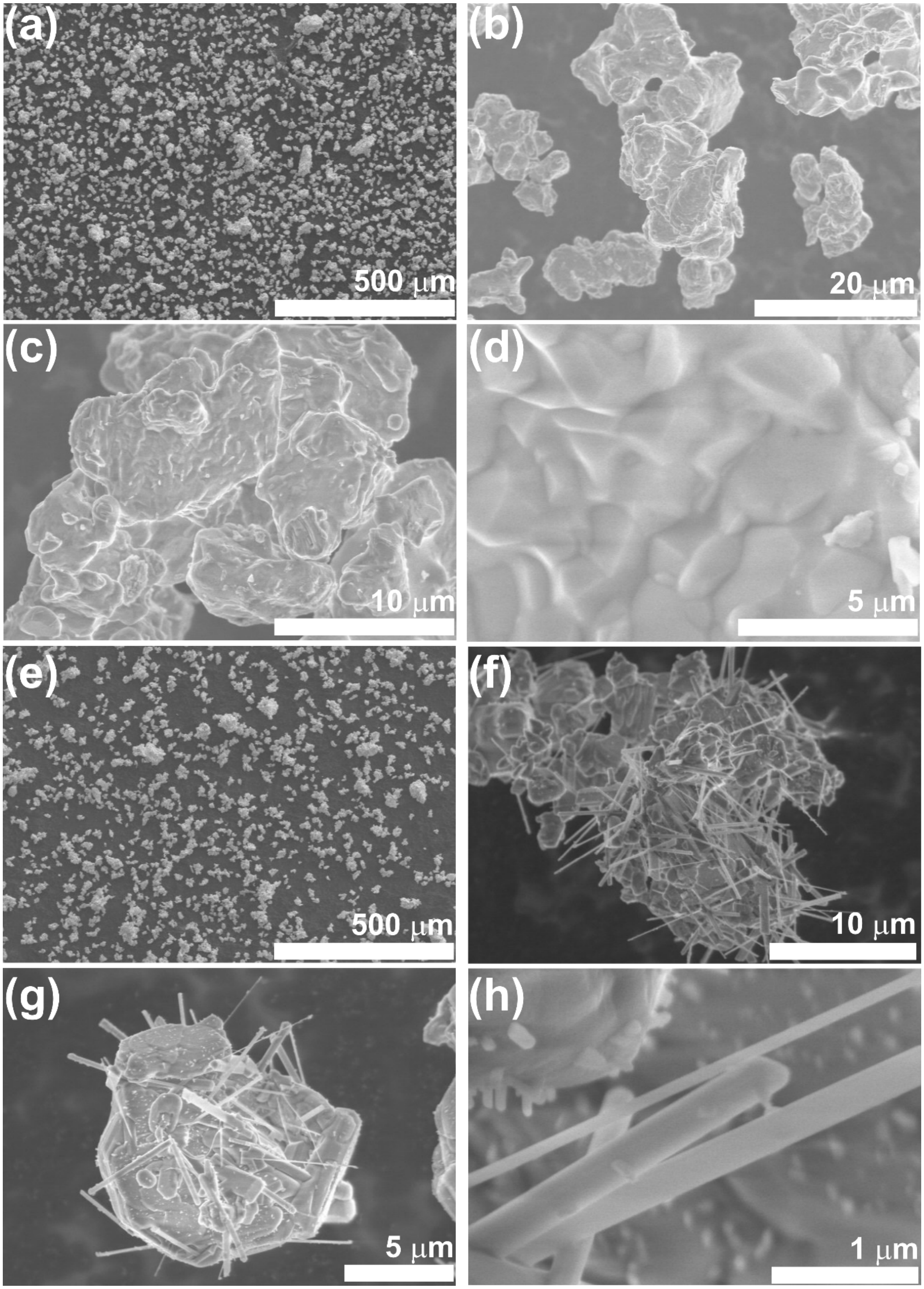

Figure S2. SEM images of (a-d) GO 1200 phospho and (e-h) GO 1350 phosphor.

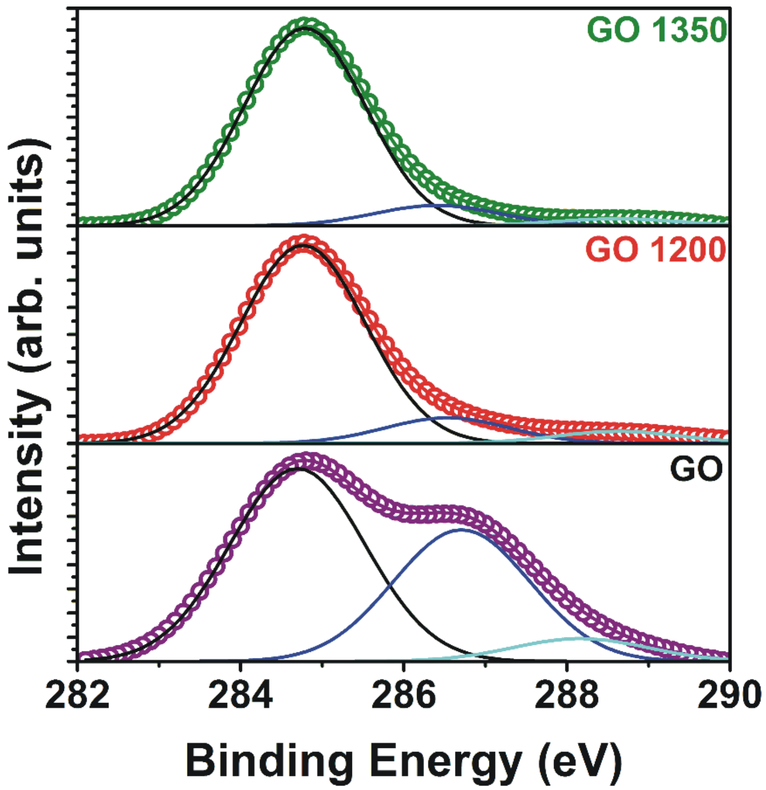

Figure S3. High-resolution C *1s* XPS data of GO, GO 1200, and GO 1350.

The C *1S* high-resolution XPS data (Fig. S3) for GO exhibit peaks corresponding to sp^2 carbon at 284.8 eV, O-H/O-C-O at 286.7 eV, C=O at 287.8 eV, O-C=O at 288.5 eV and COOH groups at 289 eV.[1,2] However, the spectra from GO, GO 1200, and GO 1350 show considerable reduction of oxygenated carbon signals. The XPS intensities related to oxygenated functional groups were reduced because of the annealing under a reducing atmosphere. Moreover, no other peaks related to carbon bonding with the elements in the phosphor (Sr, Ba, or Si) are observed. It is concluded from the XPS data that reduction of GO occurs when annealed at high temperatures at and above 1200 °C.

The Sr *3d* and O *1s* high-resolution XP spectra of fresh, unwrapped, and GO-wrapped phosphors are shown in Figure S3. The Sr *3d* and O *1s* spectra of UW1350 and GO 1350 do not show significant variation with annealing or GO wrapping while Si *2p* and N *1s* exhibit minor shifts in the binding energy.

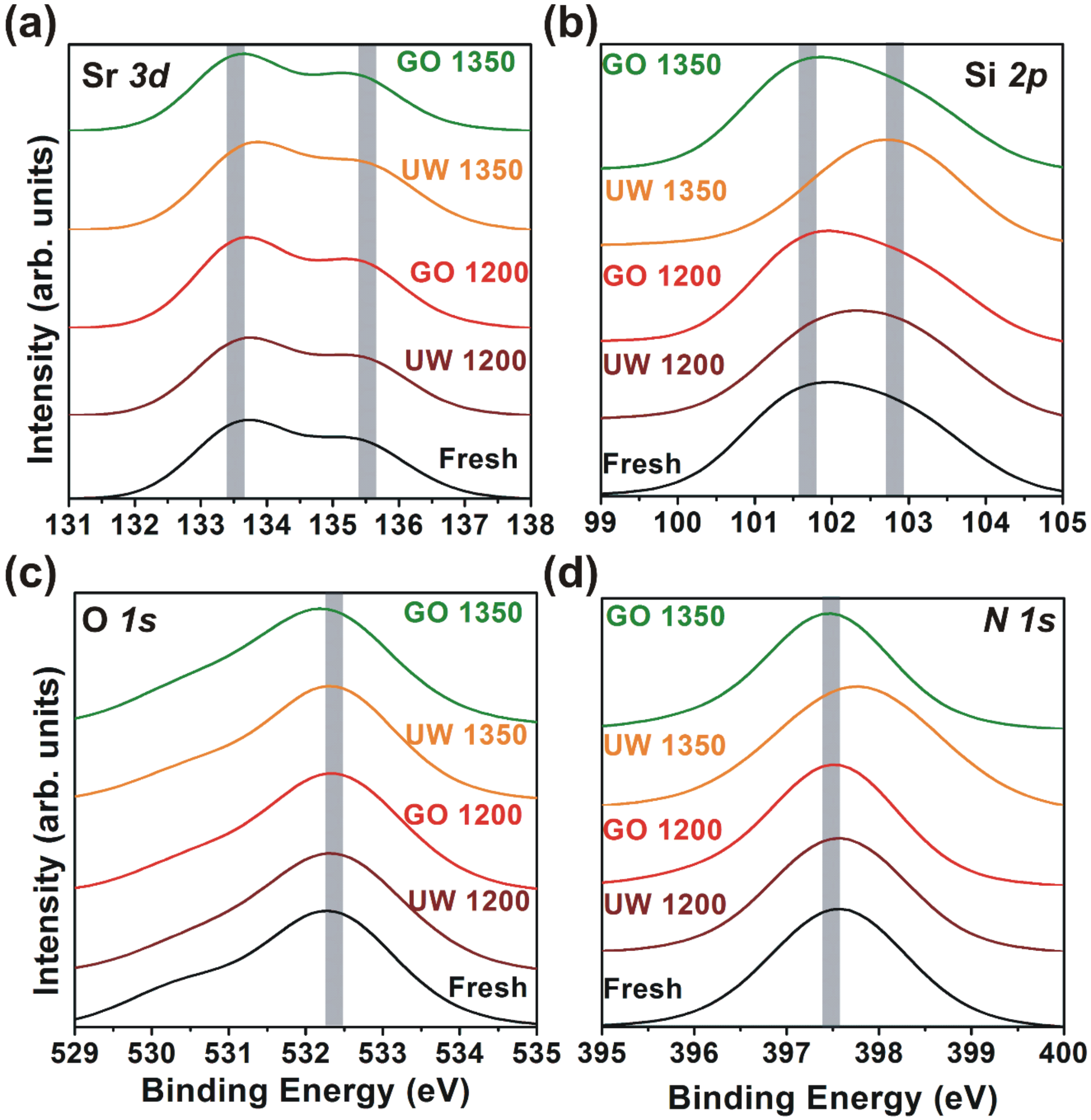

Figure S4. High-resolution XPS data from unwrapped and wrapped phosphors: (a) Sr *3d*, (b) Si *2p*, (c) O *1s*, and (d) N *1s*.

These shifts are a result of bonding of rGO sheets with the phosphor particle through C-N bonding. The formation of nanoscrolls presumably occurs through this bonding. It is concluded from the XPS analyses that C in rGO bonds with N in the phosphor in GO 1200 and GO 1350 phosphor samples.

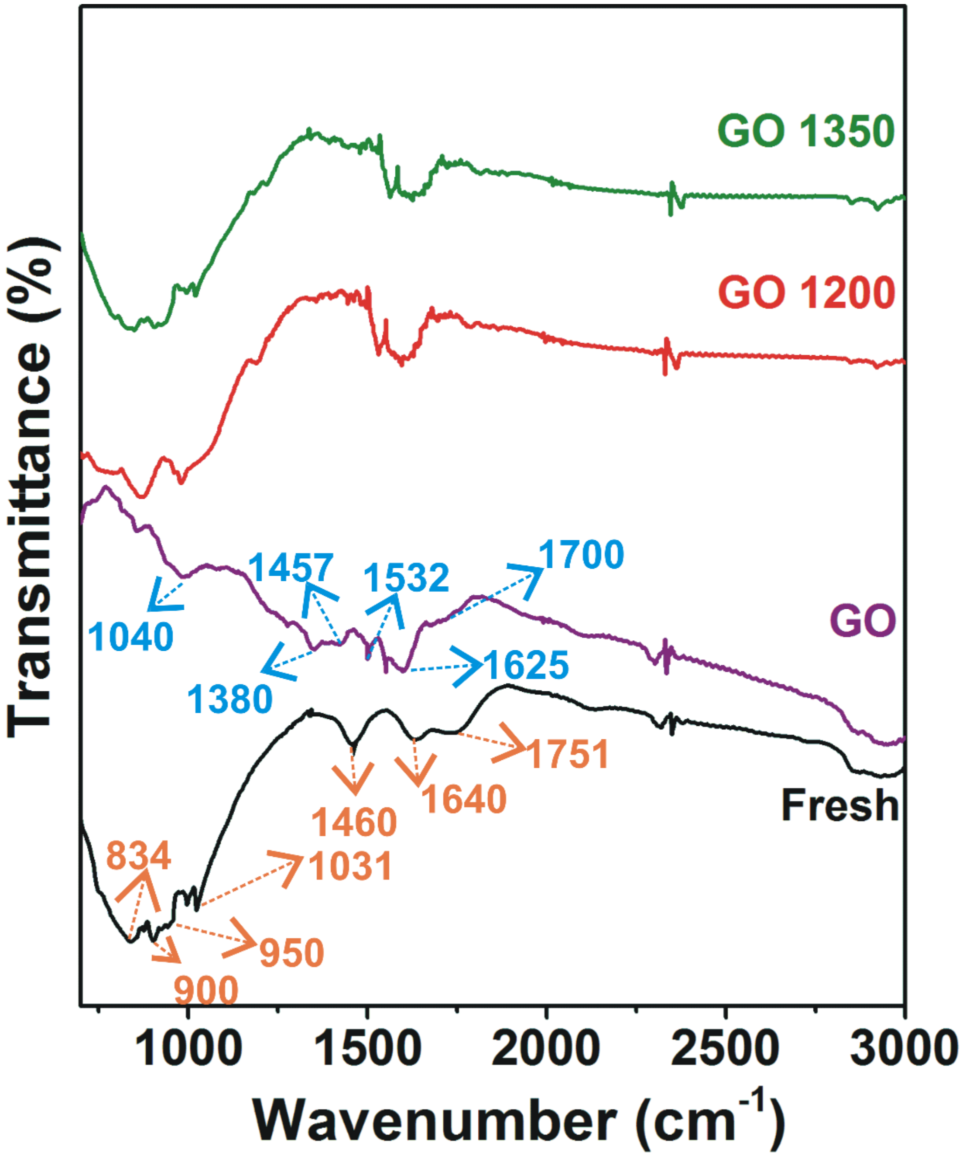

Figure S5. The FTIR spectra of fresh phosphor, GO, GO 1200, and GO 1350.

In the FTIR spectrum from GO, peaks are observed at 1040 cm^{-1} (C–C vibrations)[3], 1130 cm^{-1} (C–O–C stretching)[4], 1380 cm^{-1} (O–H in-plane bending)[5], 1457 cm^{-1} (C–C ring stretching) [5],1530 (CH_2 bending) [6], 1582 cm^{-1} (conjugated C=C stretching) [5], 1625 cm^{-1} (non-conjugated C=C stretching vibrations) [5], and 1720 cm^{-1} (C=O stretching). [5,7] The vibrations observed around 800-900 cm^{-1} correspond to various C-O vibrations.[7] The peaks corresponding to C-O-C vibrations, O-H in-plane bending, CH_2 bending, and C=O stretching are absent in GO 1200 and GO 1350, which apparently indicates the removal of oxygen functional groups due to annealing. The peak around 1040 cm^{-1} (C-C) observed in GO shifts to 1070 cm^{-1} in GO 1200 and in GO 1350 phosphors. Additionally, the C-C ring stretching vibration[5] observed at 1457 cm^{-1} in GO shifts to 1438 cm^{-1} in GO 1200 and GO 1350 samples. The conjugated C=C stretching vibration also shifts from 1582 cm^{-1} to 1570 cm^{-1} in GO 1200 and GO 1350. The observed shifting of these peaks is apparently due to the attachment of phosphor particles to the GO sheets because of the high temperature annealing. However, non-conjugated C=C stretching vibrations at 1625 cm^{-1} remain the same in GO, GO 1200, and GO 1350 samples.

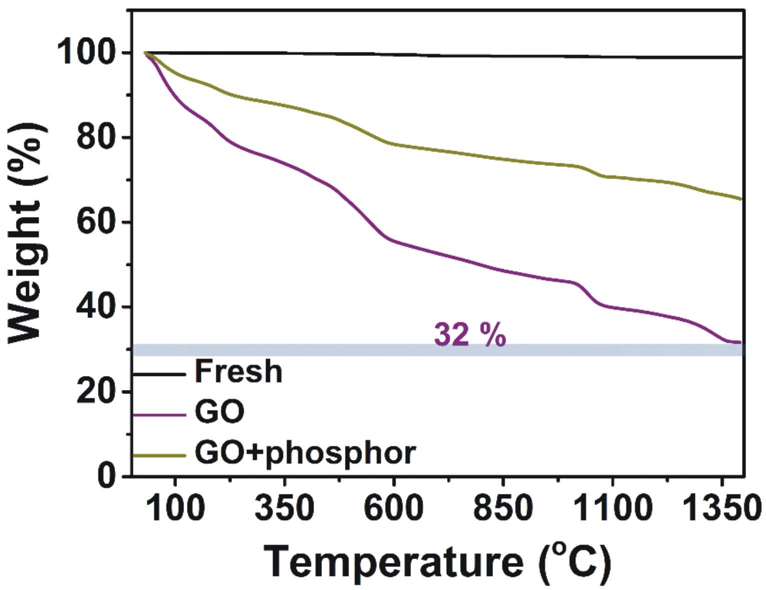

Figure S6. The TGA of fresh phosphor, GO, and GO-phosphor composite (1:1 weight ratio)

To study the stability of GO in the GO-phosphor composite at high temperatures, TG thermograms of the GO and fresh phosphor were recorded and analyzed. As shown in Fig. S6, in GO, the major weight loss occur between 100 and 200 °C, between 350 and 500 °C and again around 900 °C. The weight loss below 200 °C is due to the removal of water and oxidation of carbon to CO_2.[8] Above 200 °C, the weight loss occurs because of the removal of oxygen functional groups.[8] The fresh phosphor is stable throughout the temperature range. The GO-phosphor composite also shows behavior similar to that of GO in which the weight loss is attributed to the removal of water and functional groups in GO.

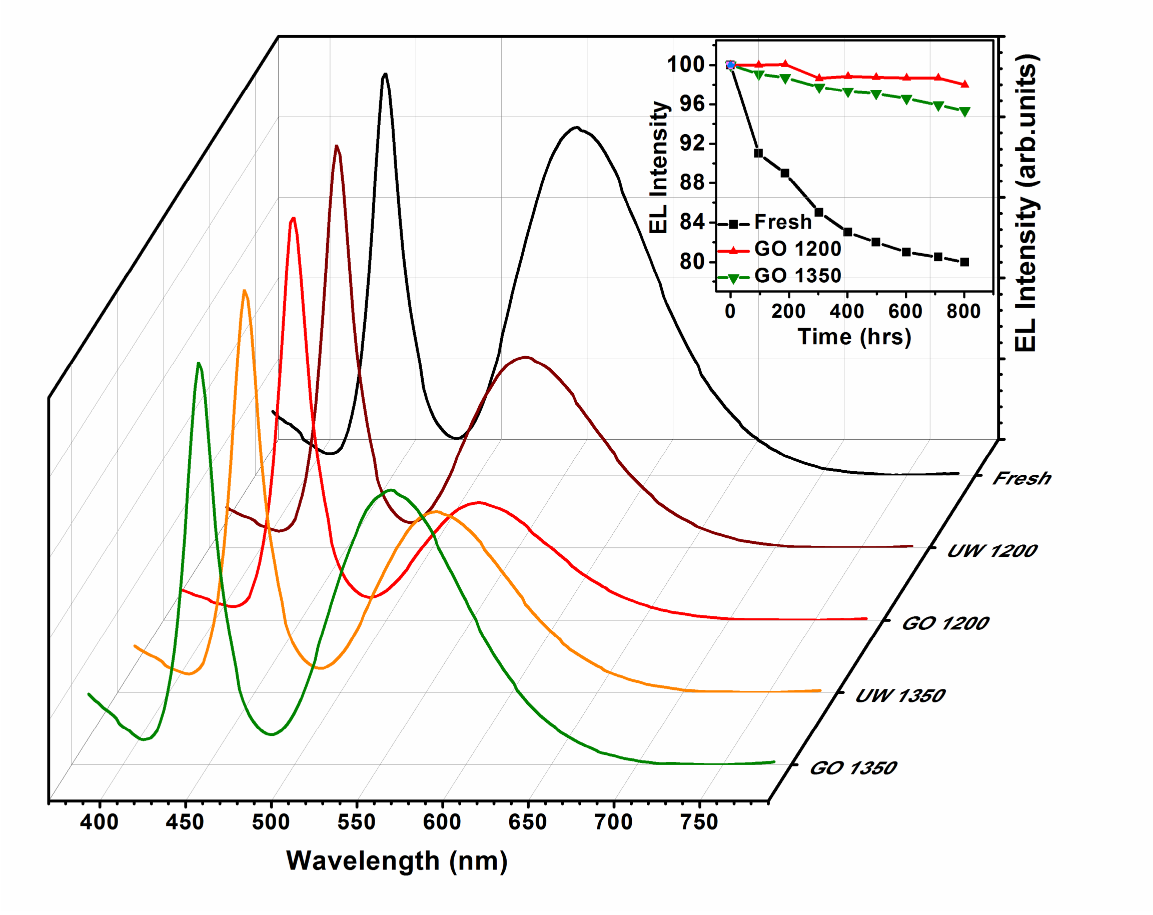

Figure S7. The EL emission from the pcLED package fabricated using various phosphors, inset shows the variation of EL intensities of pcLEDs fabricated using fresh, GO 1200, and GO 1350 phosphors, operated continuously at 85 ^{o}C and 85 % rel. humidity.

The EL emission from the pcLEDs fabricated using unwrapped and wrapped phosphors are shown in Fig. S7. The inset shows the reliability test data of fresh, GO 1200 and GO 1350 phosphors. As seen in the inset, the pcLEDs fabricated using GO 1350 phosphor exhibit rapid degradation as compared to pcLEDs fabricated using GO 1200 phosphor. This is due to the rGO nanoscrolls formation in GO 1350 phosphors leading to poor heat dissipation.

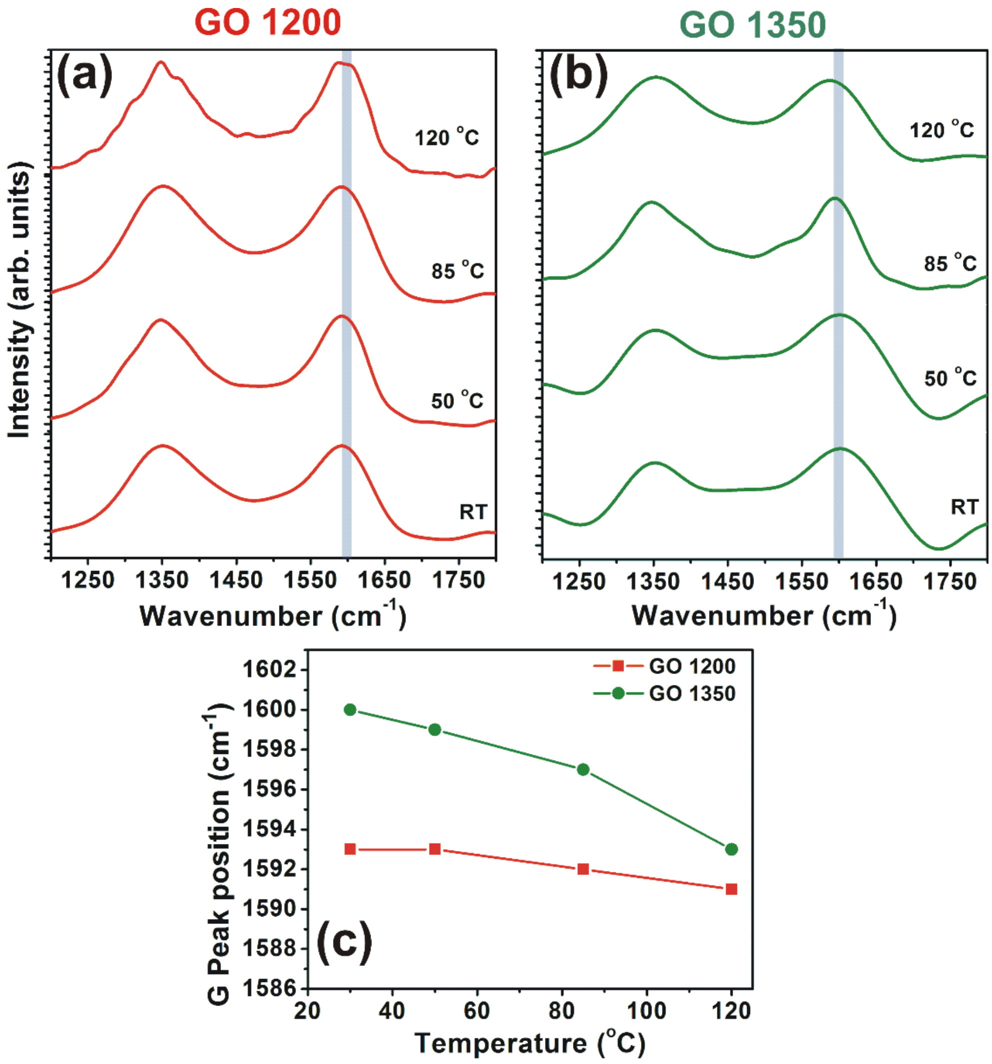

Figure S8. The Raman spectra of (a) GO 1200 and (b) GO 1350 phosphors at various temperatures (RT-room temperature). (c) The Raman G peak shift as a function of the temperature.

The Raman spectra of GO 1200 and GO 1350 phosphor samples at various temperatures are shown in Fig. S8(a) and (b) respectively. The G peak shift as a function of temperature is shown in Fig. S8(c). It is apparent from the spectra that the G peak red shifts with an increase in the temperature. The variation of G peak position with temperature can be represented as

$$\omega = \omega_0 + \chi T \qquad (1)$$

where ω_0 is the G peak position at 0 K, χ is the first order temperature coefficient and T is the absolute temperature.[9] The variation in the phonon frequency is mainly attributed to the anharmonic coupling of phonon modes and thermal expansion of the lattice. [9,10] The G peak position of GO 1200 phosphor does not show significant variation in the investigated temperature range whereas GO 1350 exhibit considerable shift in G peak position. This once again supports our conclusion that the formation of nanoscrolls in GO 1350 limits the heat dissipation.

References

1 Rani, J. R. *et al.* Graphene oxide–phosphor hybrid nanoscrolls with high luminescent quantum yield: Synthesis, structural, and X-ray absorption studies. *ACS Appl. Mater. Interfaces* **7**, 5693-5700 (2015).

2 Rani, J. R. *et al.* Epoxy to carbonyl group conversion in graphene oxide thin films: Effect on structural and luminescent characteristics. *J. Phys. Chem. C* **116**, 19010-19017 (2012).

3 Bosch-Navarro, C., Coronado, E., Marti-Gastaldo, C., Sanchez-Royo, J. F. & Gomez, M. G. Influence of the pH on the synthesis of reduced graphene oxide under hydrothermal conditions. *Nanoscale* **4**, 3977-3982 (2012).

4 Bhowmik, K., Pramanik, S., Medda, S. K. & De, G. Covalently functionalized reduced graphene oxide by organically modified silica: a facile synthesis of electrically conducting black coatings on glass. *J. Mater. Chem.* **22**, 24690-24697 (2012).

5 Rani, J. R. *et al.* Controlling the luminescence emission from palladium grafted graphene oxide thin films via reduction. *Nanoscale* **5**, 5620-5627 (2013).

6 Navarro-Pardo, F. *et al.* Effects on the Thermo-Mechanical and Crystallinity Properties of Nylon 6,6 Electrospun Fibres Reinforced with One Dimensional (1D) and Two Dimensional (2D) Carbon. *Materials* **6** (2013).

7 Aunkor, M. T. H., Mahbubul, I. M., Saidur, R. & Metselaar, H. S. C. The green reduction of graphene oxide. *RSC Adv.* **6**, 27807-27828 (2016).

8 Feng, H., Cheng, R., Zhao, X., Duan, X. & Li, J. A low-temperature method to produce highly reduced graphene oxide. *Nat. Commun.* **4**, 1539 (2013).

9 Calizo, I., Balandin, A. A., Bao, W., Miao, F. & Lau, C. N. Temperature dependence of the Raman spectra of graphene and graphene multilayers. *Nano Lett.* **7**, 2645-2649 (2007).

10 Sahoo, S. *et al.* In situ Raman studies of electrically reduced graphene oxide and Its field-emission properties. *J. Phys. Chem. C* **117**, 5485-5491 (2013).

Appendix

The XRD peaks and their labels according to PDF-01-076-3141

2θ	h	k	l	2θ	h	k	l
12.241	0	1	0	28.941	-1	0	2
12.612	0	1	0	28.941	-1	-2	1
12.933	1	0	0	29.872	-2	-1	1
15.739	1	1	0	29.872	-2	0	1
17.024	1	0	1	30.337	-1	-1	2
17.603	0	1	1	31.106	1	-1	2
17.603	0	-1	1	31.34	-1	2	0
18.629	-1	0	1	31.746	2	2	0
19.262	1	1	1	31.746	-2	1	0
20.2	-1	1	0	32.976	-1	1	2
20.687	-1	-1	1	33.308	1	-2	1
23.061	1	-1	1	33.308	2	2	1
24.296	-1	1	1	34.438	2	1	2
24.625	0	0	2	34.438	2	0	2
25.381	0	2	0	34.956	-2	1	1
25.561	1	2	0	34.956	1	2	2
26.035	2	1	0	39.59	0	1	3
26.035	2	0	0	39.952	-1	-3	1
26.841	1	0	2	40.376	3	0	1
27.747	0	1	2	40.517	0	3	1
27.747	0	-1	2	40.517	0	-3	1
27.921	1	2	1	41.115	2	3	0
28.351	1	1	2	41.115	-2	2	0
28.351	0	2	1	41.681	1	-1	3

41.681	3	2	0	47.206	2	3	2
41.828	-1	-1	3	47.476	-3	1	1
42.08	-2	-2	2	47.809	2	2	3
42.08	-2	1	2	47.809	2	-1	3
42.502	3	2	1	48.164	-3	-1	2
42.6	-3	0	1	48.503	3	3	0
43.826	-1	1	3	48.503	1	-2	3
43.826	-1	3	0	49.078	-3	0	2
43.976	2	1	3	49.263	3	3	1
43.976	2	0	3	49.78	-2	-3	2
44.088	3	1	2	49.78	-2	2	2
44.64	1	3	2	50.489	0	0	4
44.64	-3	1	0	50.489	-1	2	3
44.755	1	2	3	50.898	-3	-2	2
45.052	3	0	2	51.097	1	0	4
45.322	1	-3	1	51.163	-3	-3	1
45.436	3	-1	1	51.561	-1	3	2
45.684	0	2	3	51.829	1	4	1
45.684	0	-2	3	51.829	4	1	0
46.036	-1	-3	2	51.995	1	1	4
46.036	-1	3	1	52.126	0	4	0
46.24	0	3	2	52.247	0	1	4
46.24	0	-3	2	52.247	0	-1	4
46.773	-1	-2	3	52.431	-1	-4	1
46.773	-1	-2	3	52.519	2	4	0
47.013	3	2	2	52.519	-2	3	0
47.129	2	-2	2	52.724	3	0	3

52.906	-3	2	0	60.38	-4	-1	2
53.033	1	3	3	60.38	-3	-4	1
53.337	3	3	2	60.902	-3	2	2
55.855	4	1	2	61.233	-4	1	1
56.123	-1	1	4	61.78	-1	2	4
56.277	-4	0	1	62.059	-3	1	3
56.277	1	2	4	62.059	-4	-2	2
56.366	-4	-2	1	62.264	3	4	2
56.366	1	4	2	62.264	4	-1	2
56.933	3	-1	3	62.654	1	-4	2
56.933	-3	-3	2	62.654	3	0	4
57.314	0	2	4	63.109	-2	1	4
57.314	0	-2	4	63.109	-2	-2	4
57.532	4	2	2	63.483	1	3	4
57.532	4	0	2	63.483	-3	3	0
57.604	2	-3	2	63.62	1	4	3
57.698	2	4	2				
58.016	3	4	0				
58.016	1	-3	3				
58.49	0	4	2				
58.49	0	-4	2				
58.652	2	2	4				
58.652	4	3	0				
59.483	1	-2	4				
59.935	-2	0	4				
59.935	-2	3	2				
60.253	3	3	3				
